# Myocardial electrotonic response to submaximal exercise in dogs with healed myocardial infarctions: evidence for β-adrenoceptor mediated enhanced coupling during exercise testing

**DOI:** 10.3389/fphys.2015.00025

**Published:** 2015-02-05

**Authors:** Carlos L. del Rio, Bradley D. Clymer, George E. Billman

**Affiliations:** ^1^Department of Physiology and Cell Biology, The Ohio State UniversityColumbus, OH, USA; ^2^Department of Electrical and Computer Engineering, The Ohio State UniversityColumbus, OH, USA; ^3^Safety Pharmacology, QTest LabsColumbus, OH, USA; ^4^Biomedical Engineering, The Ohio State UniversityColumbus, OH, USA; ^5^Davis Heart and Lung Research Institute, The Ohio State UniversityColumbus, OH, USA

**Keywords:** electrotonic coupling, β-adrenoceptor stimulation, exercise, arrhythmic risk, myocardial infarction

## Abstract

**Introduction:** Autonomic neural activation during cardiac stress testing is an established risk-stratification tool in post-myocardial infarction (MI) patients. However, autonomic activation can also modulate myocardial electrotonic coupling, a known factor to contribute to the genesis of arrhythmias. The present study tested the hypothesis that exercise-induced autonomic neural activation modulates electrotonic coupling (as measured by myocardial electrical impedance, MEI) in post-MI animals shown to be susceptible or resistant to ventricular fibrillation (VF).

**Methods:** Dogs (*n* = 25) with healed MI instrumented for MEI measurements were trained to run on a treadmill and classified based on their susceptibility to VF (12 susceptible, 9 resistant). MEI and ECGs were recorded during 6-stage exercise tests (18 min/test; peak: 6.4 km/h @ 16%) performed under control conditions, and following complete β-adrenoceptor (β-AR) blockade (propranolol); MEI was also measured at rest during escalating β-AR stimulation (isoproterenol) or overdrive-pacing.

**Results:** Exercise progressively increased heart rate (HR) and reduced heart rate variability (HRV). In parallel, MEI decreased gradually (enhanced electrotonic coupling) with exercise; at peak exercise, MEI was reduced by 5.3 ± 0.4% (or -23 ± 1.8Ω, *P* < 0.001). Notably, exercise-mediated electrotonic changes were linearly predicted by the degree of autonomic activation, as indicated by changes in either HR or in HRV (*P* < 0.001). Indeed, β-AR blockade attenuated the MEI response to exercise while direct β-AR stimulation (at rest) triggered MEI decreases comparable to those observed during exercise; ventricular pacing had no significant effects on MEI. Finally, animals prone to VF had a significantly larger MEI response to exercise.

**Conclusions:** These data suggest that β-AR activation during exercise can acutely enhance electrotonic coupling in the myocardium, particularly in dogs susceptible to ischemia-induced VF.

## Introduction

Myocardial infarction is a well-established risk factor for sudden cardiac death (SCD) due to malignant arrhythmias (Adabag et al., 2010; Zaman and Kovoor, 2014). However, despite significant advances in the understanding of the physiological substrate(s) mediating/facilitating the onset of arrhythmias, risk stratification for SCD in post-MI patients remains difficult and insufficient (Goldberger et al., 2014; Wellens et al., 2014; Zaman and Kovoor, 2014). Indeed, the majority of SCD episodes occur in patients with either low-/intermediate- or without known risk factors (e.g., Wellens et al., 2014).

In these patients, underlying ionic current abnormalities, particularly those mediating repolarization, either co-exist with and/or are exacerbated by autonomic imbalances favoring enhanced sympathetic drive (e.g., Chen et al., 2007; Pokornı et al., 2011; Wellens et al., 2014). As such, multi-modality stratifications techniques, encompassing electrocardiographic evaluation of repolarization abnormalities during states of autonomic activation, such as exercise, are favored (Goldberger et al., 2014; Wellens et al., 2014). For instance, the assessment of microvolt T-wave alternans (TWA or MTWA) during low-intensity exercise has been shown to predict not only arrhythmic events in post-MI patients but also arrhythmia-free survival in patients with LV dysfunction (Cantillon et al., 2007; Amit et al., 2010; Verrier et al., 2011; Merchant et al., 2012; Shizuta et al., 2012).

Interestingly, activation of the autonomic nervous system (e.g., during exercise), and its concomitant catecholamine release, may in turn also modulate the passive electrical properties that govern electrotonic interactions in the myocardium. For example, several studies have shown that catecholamines (and increased cAMP levels) enhance junctional coupling in myocytes (e.g., see De Mello, 1996a; Dhein, 2004; Salameh and Dhein, 2011). Notably, electrotonic coupling is a well-established factor modulating both repolarization disturbances and arrhythmic risk, as poorly coupled cells are more likely to exhibit pro-arrhythmic behaviors (e.g., Pastore and Rosenbaum, 2000; De Groot and Coronel, 2004; Saffitz and Kléber, 2012; Wit and Peters, 2012). For instance, enhanced electrotonic coupling has been shown to suppress early after-depolarizations (EADs) (Huelsing et al., 2000; Himel et al., 2013), and reduce transmural dispersion of repolarization (Quan et al., 2007). Similarly, preserved electrotonic interaction has been shown to modulate TWA *in silico*, and more recently, also *in vivo* (Pastore and Rosenbaum, 2000; Watanabe et al., 2001; Cherry and Fenton, 2004; Sato et al., 2006; Kjølbye et al., 2008; Jia et al., 2012). Remarkably, no study to date has investigated concomitant passive electrical (electrotonic) changes during autonomic neural activation *in vivo*.

It was, therefore, the purpose of this study to investigate myocardial electrotonic coupling changes induced by submaximal exercise in the left-ventricle of post-MI animals, as measured by myocardial electrical impedance (MEI). Specifically, the hypothesis that exercise-induced autonomic activation can modulate myocardial electrotonic coupling (i.e., MEI) was tested in animals with healed myocardial infarctions later demonstrated to be either susceptible or resistant to ischemia-induced VF. Briefly, β-adrenoceptor (β-AR) activation during submaximal exercise acutely decreased the electrical impedance of the surviving myocardium, particularly in animals susceptible to VF, consistent with an increased electrotonic coupling.

## Materials and methods

The principles governing the care and treatment of animals, as expressed by the American Physiological Society, were followed at all times during this study. In addition, the animal protocols and experimental procedures were approved by The Ohio State University's Institutional Lab Animal Care and Use Committee (ILACUC) at this institution, and adhered to the statutes of the Animal Welfare Act and the guidelines of the Public Health Service.

### Surgical preparation

The studies were performed using a well-characterized canine model of sudden cardiac death, known to mimic/combine the most prevalent features associated with this disease in the clinic: healed myocardial ischemic injury, acute myocardial ischemia, and cardiac autonomic activation (see Billman, 2006).

Briefly, thirty-five (*n* = 35) heartworm-free purpose bred mixed-breed dogs (weight: 16.1–24.1 kg, 19.0 ± 0.4 kg) were sedated (morphine sulfate 15 mg IM, and thiopental sodium 20 mg/kg IV), and connected to a respirator via an endotracheal cuffed tube. Anesthesia was maintained with inhaled isoflurane (1–1.5%) mixed with oxygen (100%). Under sterile conditions, the chest was opened via a left thoracotomy (fifth intercostal space); the heart was exposed, and suspended with a pericardial cradle. Subsequently, an antero-lateral myocardial infarction (MI) was created by a two-stage ligature of the left anterior descending (LAD) coronary artery. The left circumflex (LCX) coronary artery was dissected free of the surrounding tissue near its origin (under the edge of the left atrial appendage) and was instrumented with a 20 MHz Doppler-flow transducer, and a hydraulic coronary artery occluder; inflation of this balloon would later render a portion of the LCX distribution acutely ischemic (see *Arrhythmia Susceptibility*).

As required for MEI measurements (see below), a bipolar pacing electrode (Medtronic Inc., model Streamline™ 6495) was placed remote to the infarct, in the distal (non-ischemic) distribution of the LCX coronary artery. In a subset of animals (*n* = 10), a second MEI electrode was placed in the healthy (*non-infarcted*) anterior myocardium for pacing purposes (see *Experimental Protocol*). The leads were inserted into the mid-myocardial wall (parallel to the local fiber alignment), and were firmly secured in place with non-absorbable sutures (prolene 2-0). The pericardial cradle was released, the chest closed in layers and evacuated of air restoring the negative intra-thoracic pressure. All leads were tunneled under the skin, exited at the neck, and were carefully bandaged.

### Exercise test protocol

**Figure 1:**
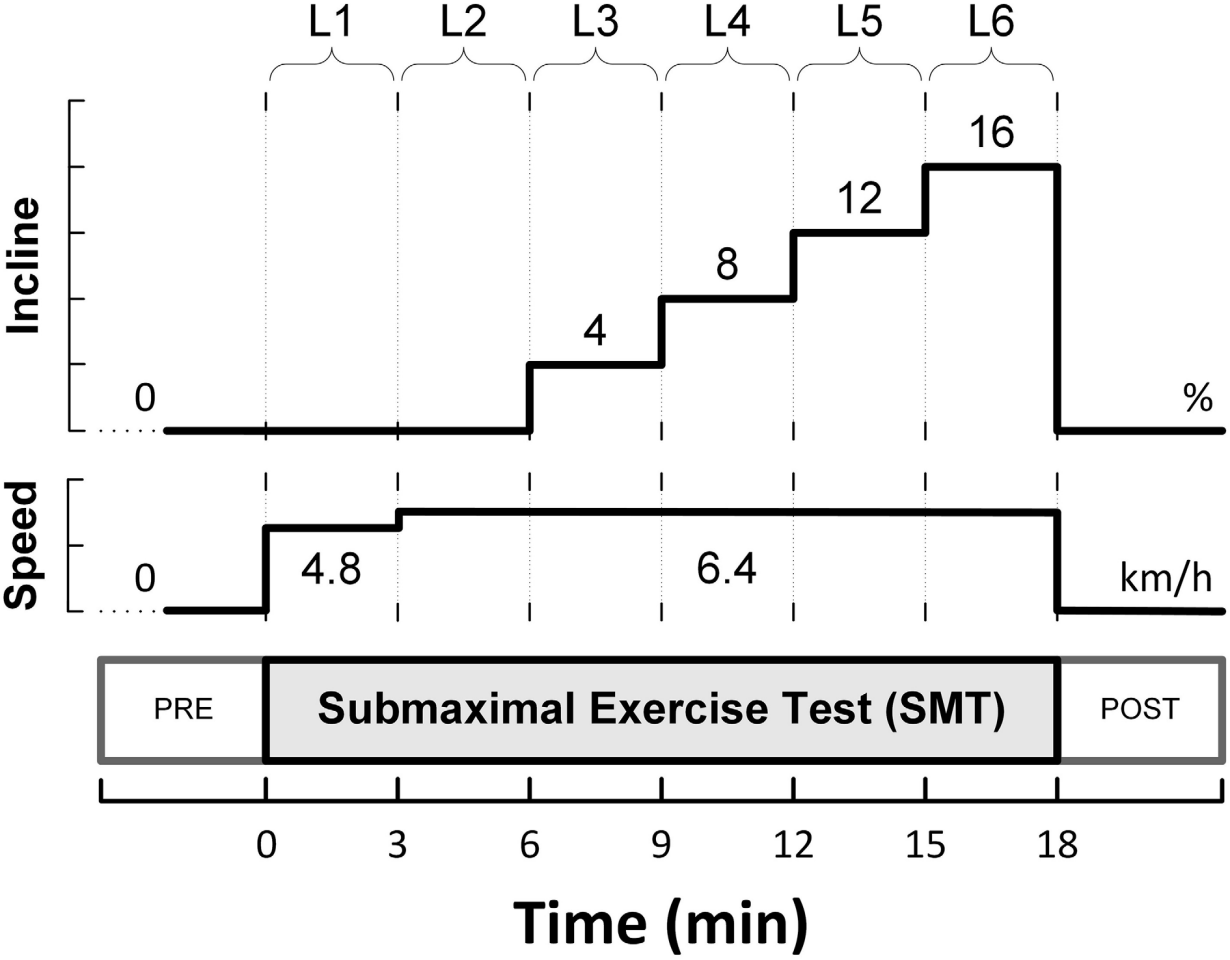
**Schematic representation of the six-level submaximal exercise test (SMT)**.

The animals were allowed to recover for 3–4 weeks, and subsequently, were trained to run on a motor-driven treadmill. A 6-stage submaximal exercise stress-test (SMT), as initially described by Stone (1977), was used to activate the autonomic nervous system. This protocol is summarized in Figure 1, and consisted of a 3 min warm-up walking period (4.8 km/h, 0% grade; level L1), followed by running (6.4 km/h) for 15 min with the grade (incline) increased every 3 min (i.e., 0, 4, 8, 12, and 16%; levels L2-L6).

### Arrhythmia susceptibility

The susceptibility to ischemia-induced ventricular fibrillation was assessed at the end of the study using a standardized protocol, generally referred as the “exercise-plus-ischemia” test (see Billman, 2006). In short, a submaximal exercise bout was performed (as described above) and during the last minute of exercise, the animals were subjected to a brief (2 min) LCX occlusion (i.e., while running at 6.4 km/h, 16%). This combination of exercise plus ischemia, when applied post-MI, yields two stable and well-differentiated populations of animals: one susceptible and the other resistant to ischemia-induced malignant arrhythmias, such as ventricular fibrillation [see Billman (2006) for review]. In this study, 12 animals developed VF (susceptible, S) and 9 did not (resistant, R) during the exercise-plus-ischemia test. Four animals (*n* = 4) could not be classified due to equipment failure (e.g., occluder rupture).

### Myocardial electrical impedance (MEI)

As has previously been described, a computer controlled circuit developed in this laboratory was used to measure the complex electrical impedance of the myocardium (Howie et al., 2001; Dzwonczyk et al., 2004; Del Rio et al., 2005, 2008a,b). In short, using a bipolar pacing lead (see above) the myocardium was probed with a sub-threshold zero-mean bipolar current, consisting of two rectangular pulse of alternating polarity (± 5 μA, 100 μs wide) generated 200 ms apart. The complex MEI spectrum was calculated in the frequency domain, as the ratio (at each frequency) of the current and voltage spectra resulting from the ensemble averages of 10 stimulus pulses and their respective (voltage) responses. The mean modulus of the complex MEI spectrum in the 0.27–5.90 kHz frequency range was examined (Del Rio et al., 2008a).

### Experimental protocol

As described above, thirty-five animals (*n* = 35) were instrumented with MEI electrodes in the remote, non-infarcted myocardium. However, five animals (*n* = 5) experienced lead malfunctions (e.g., dislodgement) either before or at the time of experimentation, and therefore, were excluded from the analysis, while another five animals (*n* = 5) failed to acclimatize to the treadmill exercise protocol. Thus, the studies were performed in 30 animals (*n* = 30), with exercise-data successfully collected and analyzed in 25 dogs (*n* = 25).

First, in order to investigate the time-course of the electrotonic coupling (i.e., MEI) during submaximal exercise, all animals, regardless of arrhythmias susceptibility (9 resistant, 12 susceptible, and 4 unable to be classified), had MEI measurements collected during a submaximal exercise test (SMT) performed approximately 1-month after the LAD ligature (28 ± 1.7 days post-MI).

On a different day (26 ± 1.7 days post-MI), a subset of animals (5 resistant, 7 susceptible, and 4 unable to be classified; *n* = 16) performed the submaximal exercise test, but after pretreatment with the β-adrenoceptor antagonist propranolol HCl (1.0 mg/kg IV, Sigma Chemical, St. Louis, MO). Previous studies demonstrated that this dose of propranolol (1) completely abolished the cardiac response to the β-adrenoceptor agonist isoproterenol HCl (1 μg/kg IV) (Collins and Billman, 1989), and (2) did not compromise the exercise capacity during the submaximal exercise test in the presence of a 1-month-old anterior wall myocardial infarction (Brice and Stone, 1986). Propranolol was given intravenously (cephalic vein) as a bolus injection 3 min before the onset of exercise. A partially counter-balanced design was used: some dogs (6/16) were first exercise-tested under the influence of this β-adrenoceptor antagonist, and on a later day, had a control test (i.e., with no drug) performed; whereas in the remaining animals (10/16) the response to the submaximal exercise test (SMT) was first studied under control conditions, and on a subsequent day, following β-adrenoceptor blockade. In all cases, MEI measurements during the exercise tests were taken (continuously) from the distal LCX distribution (remote non-ischemic region) in awake, unsedated, and otherwise unstressed, post-MI animals (in a quiet and dimly lit room).

In order to investigate further the role of exercise-induced autonomic neural activation on myocardial electrotonic coupling (MEI), the total β-adrenoceptor response was quantified at rest in some animals (*n* = 10). Briefly, the dogs were lightly sedated with acepromazine (0.5 mg/kg IM; Ft. Dodge Animal Health, Ft. Dodge, IA), and a (cephalic vein) catheter was percutaneously placed for the administration of isoproterenol HCl (Sigma Chemical, St. Louis, MO); five increasing doses of this β-adrenoceptor agonist were given: 0.005, 0.015, 0.05, 0.15, and 0.5 μg/min/kg. MEI measurements were obtained continuously during isoproterenol infusion and washout. Data are reported (averaged over 30s) when a steady-state response was achieved at each dose, and 2 min after dosing discontinuation.

Finally, the possible confounding effects of exercise-mediated heart rate changes were evaluated in another subset of dogs (*n* = 10) via overdrive left-ventricular pacing at rest. Briefly, with the animal standing on the treadmill (awake and unsedated), an impulse generator (Grass Medical Instruments, model Grass S44, via impulse-isolation unit model SIU105-B) was used to maintain ventricular rates of 180 and 210 beats/min, mimicking those observed during moderate (L2; 6.4 km/h, 0%) and peak exercise (L6; 6.4 km/h, 16%). In these animals, a second bipolar MEI/pacing electrode was placed at the time of instrumentation (see *Surgical Preparation* above); the pacing protocol was repeated from each lead (while MEI was simultaneously recorded from the other, i.e., the non-stimulating electrode), and, as similar impedance responses were obtained, the results for the two sites (leads) were combined. MEI data collected before pacing onset, and after stabilization at each pacing rate, are reported (averaged over at least a 30s interval).

#### Data analysis

A single-lead bipolar electrocardiogram (ECG) was recorded during each presentation of the submaximal exercise test. The ECG signals were band-pass filtered and digitally sampled (1 kHz)/analyzed (on-line) using a heart rate variability (HRV) monitor (Delta-Biometrics, Inc.; Urbana-Champaign, IL). Briefly, using a previously well-described (Billman and Hoskins, 1989; Billman and Dujardin, 1990) R-R interval time-series analysis technique, the heart rate (HR) mean and its variability (i.e., HRV) were determined continuously from non-overlapping 30s-segments of the ECG. Two kinds of HRV indices were studied simultaneously: (1) two measures of statistical dispersion, namely the standard deviation (RR_SD_) and the range (RR_RNG;_ longest—shortest R-R interval) of the R-R intervals within each 30 s analysis-window; and (2) an index estimating the amplitude of the respiratory sinus arrhythmia (R-R interval variability in the 0.24—1.04 Hz frequency range), or vagal tone index (VT). MEI, HR and HRV data are reported (averaged over 30 s) at eight time-points sampled before (one), during (six), and after (one) the bouts of submaximal exercise. Pre- (baseline) and post-exercise (recovery) values were taken 2 min before/after exercise onset/offset (i.e., at *t* = −2 min, and *t* = 20 min, see Figure 1A) with the animals standing on the treadmill. Meanwhile, the six exercise data-points were recorded during the last 30s of each stage in the submaximal stress protocol (i.e., L1—L6).

In addition, exercise-induced changes in the ECG morphology as well as on ECG-derived indices of the duration and heterogeneity of ventricular repolarization were evaluated in a subset of animals (*n* = 19). In short, with the aid of pattern-recognition software (ECG Auto; EMKA Technologies, France), fiducial points/intervals were determined and measured offline from two sets of thirty consecutive ECG complexes (beats), one recorded before (i.e., at rest) and the other immediately following a control submaximal exercise test (i.e., within 5 beats of stopping the treadmill). The effects of exercise on the duration of the T-wave's terminal portion (i.e., peak-to-end interval, TPE (Yan and Antzelevitch, 1998; Opthof et al., 2007) and on the QT-interval's length, as well as on the relationship between cardiac electrical systole and diastole (i.e., ratio of QT- and TQ-intervals, QT/TQ) (Fossa et al., 2007; Kijtawornrat et al., 2010) were evaluated; both absolute (QT) and rate-corrected (QTc, via van de Water's formula; Van de Water et al., 1989) QT-intervals are reported. In addition, the standard deviation of the T-wave amplitude within each 30-beat epoch (T_SD_) was calculated and used as a surrogate-marker of temporal repolarization variability (e.g., T-wave alternans; Nearing and Verrier, 2002).

All data are presented as mean ± standard error of the mean (SEM). Statistical analyses were performed with SigmaStat (Systat Software, Inc., San Jose, CA) and NCSS (NCSS, Inc., Kaysville, UT). The mean time-course of electrotonic coupling (i.e., MEI), and ECG-derived variables during the submaximal exercise tests (SMT) was evaluated using a One-Way (exercise level: baseline, L1–L6, and recovery) analysis of variance (ANOVA) with repeated measures. Intergroup comparisons (i.e., resistant vs. susceptible) were made using a Two-Way (exercise level, and group: susceptible/resistant) ANOVA with repeated measures on one factor (exercise level). Similarly, the responses to exercise, recorded under control conditions (control) and after β-adrenoceptor blockade (beta), were compared using a Two-Way (exercise level, and control/beta tests) ANOVA with repeated measures on both factors. Finally, the statistical significance of any impedance changes induced by either pacing (3 levels: baseline/two rates) and/or by isoproterenol infusion/washout (7 levels: baseline/five doses/recovery) was evaluated using One-Way ANOVA with repeated measures. The sphericity assumption (i.e., homogeneity of the covariance matrix) was verified using the Mauchley's test (NCSS, Inc.). If this assumption was not met, then a non-parametric repeated measurements ANOVA on Ranks (Friedman) test was used. In all cases, if significant *F*-values (or *Q*-values in the non-parametric case) were observed, *post-hoc* pair-wise comparisons were made using the Tukey test.

Linear regression analyses were performed as well in order to study the relationship (interaction) between exercise-induced changes in electrotonic coupling (i.e., ΔMEI) and in two indices of autonomic activation, the heart rate (ΔHR) and the vagal-tone index (ΔVT); the regression data were “centered,” i.e., deviations from each animal's mean values (over the whole exercise bout) were studied. The equality of the ΔMEI/ΔHR (and ΔMEI/ΔVT) linear models fitted to the different groups and conditions studied (susceptible vs. resistant, and control vs. β-AR blockade) was tested by multiple linear regression analysis, considering both qualitative (group) and interaction terms (i.e., simultaneously testing the differences in slope and intersect of the regression functions). For all analyses, *P* < 0.05 was considered, a priori, to be statistically significant.

## Results

### Effects of exercise

**Figure 2:**
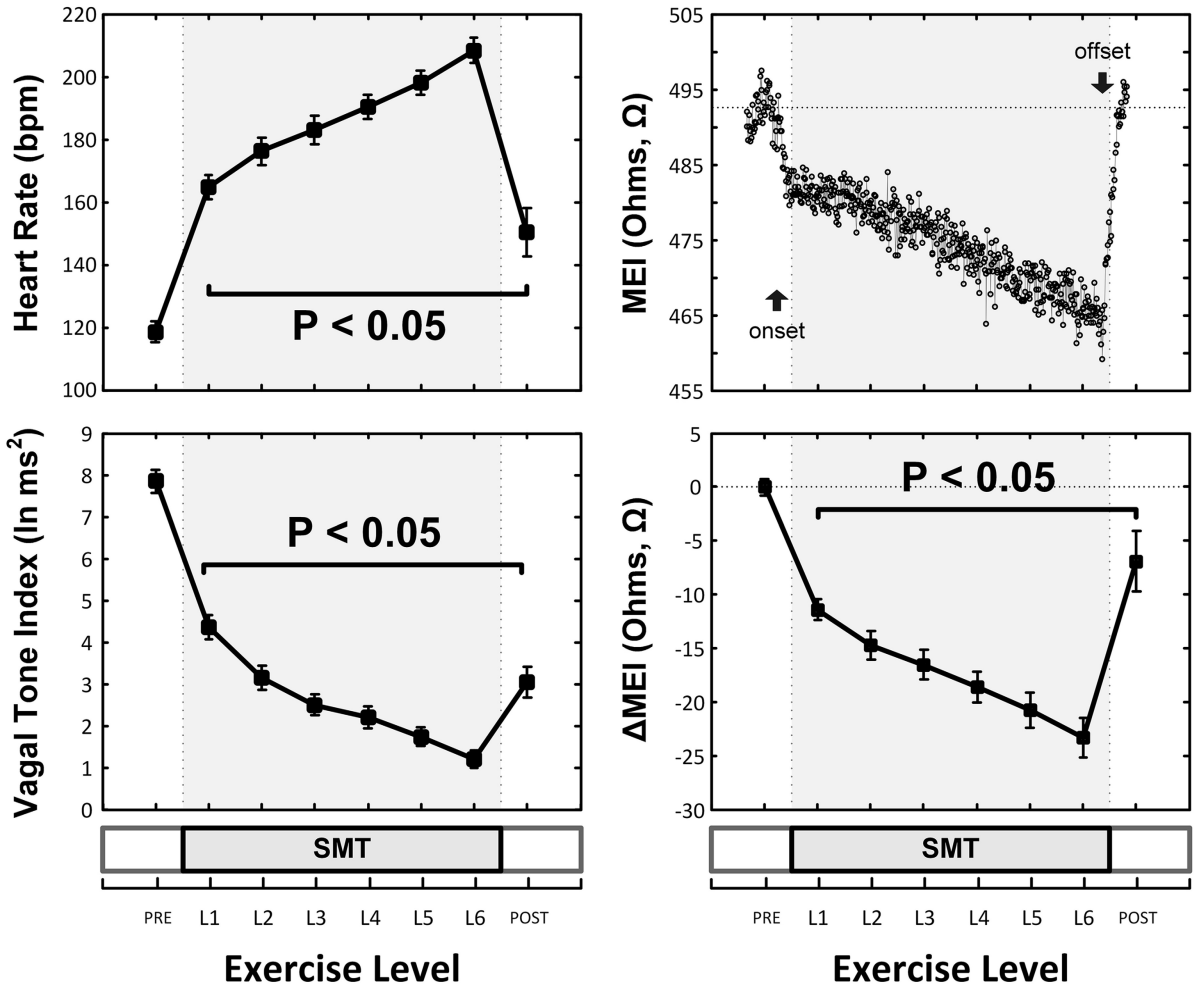
**Exercise-Induced changes in the heart-rate (HR, top-left) and ECG-derived vagal-tone index (bottom-left) and well as in the myocardial electrical impedance (MEI, bottom-right with representative response in top-right) of awake-unsedated dogs with healed left-anterior descending (LAD) myocardial infarcts (*n* = 25, except at recovery where *n* = 14)**.

As expected and consistent with previous studies (Billman and Hoskins, 1989; Billman and Dujardin, 1990; Billman, 2006), submaximal treadmill exercise resulted in a progressive acceleration of heart rate and a concomitant decrease in heart rate variability (see Figure 2 and Table 1). At peak exercise (L6; 6.4 km/h, 16%) heart rate increased on average 78 ± 4.3% (HR: from 119 ± 4 at rest to 208 ± 4 bpm at L6, *P* < 0.05), while the cardiac vagal-tone index, for instance, decreased 86 ± 2.3% (VT: from 7.9 ± 0.3 at rest to 1.2 ± 0.2 ln ms^2^ at L6, *P* < 0.05). In parallel with these changes indicative of strong cardiac autonomic neural activation, MEI decreased progressively during exercise in all animals studied (see Figure 2 and Table 1), suggesting enhanced electrotonic coupling. For example, at the highest exercise level (i.e., L6) MEI decreased −23 ± 1.8Ω (or 5.3 ± 0.4%) from the pre-exercise (at rest) values (MEI: from 446 ± 16 to 423 ± 16Ω at L6, *P* < 0.05). Moreover, following discontinuation of exercise offset (i.e., recovery), all parameters returned toward the pre-exercise (baseline) values.

**Table 1:** **Myocardial electrical impedance (MEI), heart-rate (HR) and ECG-derived indices of heart rate variability in awake-unsedated dogs with healed left-anterior descending (LAD) myocardial infarcts, both before (baseline) as well as during a submaximal exercise test**.

	**Baseline**	**Submaximal exercise test (SMT)**			**Correlation**
**Parameter**	**0 km/h, 0%**	**6.4 km/h, 0%**	**6.4 km/h, 8%**	**6.4 km/h, 16%**	**vs. ΔMEI**
MEI (Ohms)	446 ± 16	431 ± 16^*^	427 ± 16^*^	423 ± 16^*^	–
Heart rate (bpm)	119 ± 3	176 ± 4^*^	190 ± 4^*^	208 ± 4^*^	(–) *R*^2^ = 0.83
Vagal Tone (ln ms^2^)	7.9 ± 0.3	3.1 ± 0.3^*^	2.2 ± 0.3^*^	1.2 ± 0.2^*^	(+) *R*^2^ = 0.77
RR_SD_ (ms)	71 ± 6	22 ± 2^*^	14 ± 1^*^	8 ± 1^*^	n/s
RR_RNG_ (ms)	329 ± 30	101 ± 8^*^	63 ± 7^*^	38 ± 4^*^	n/s

* P < 0.05 vs. Baseline.

n/s, not studied.

**Figure 3:**
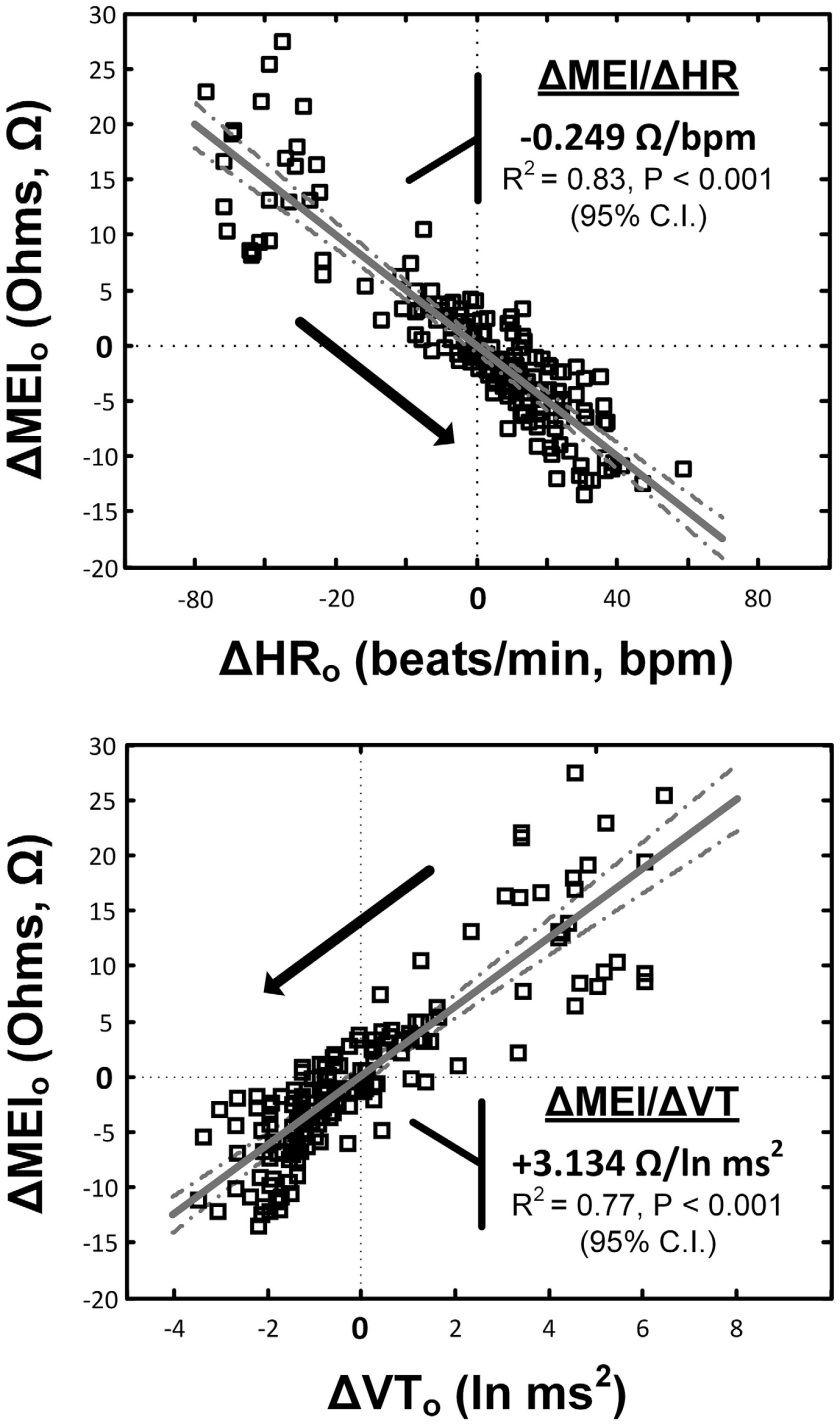
**Relationship(s) between the exercise-induced changes in the heart-rate (top) and ECG-derived vagal-tone index (bottom) with the concomitant reductions in myocardial electrical impedance (MEI); relationships were “centered,” i.e., deviations from each animal's mean values (over the whole exercise bout) were studied**.

Notably, exercise-mediated impedance changes (ΔMEI) were linearly predicted by (i.e., correlated with) the degree of autonomic neural activation, as indicated by either changes in heart rate (ΔHR; slope ΔMEI vs. ΔHR = −0.249 Ω /bpm; *R*^2^ = 0.83, *P* < 0.05), or in vagal tone index (ΔVT; slope ΔMEI vs. ΔVT = 3.134 Ω /ln(ms^2^); *R*^2^ = 0.77, *P* < 0.05) (see Figure 3 and Table 1).

**Figure 4:**
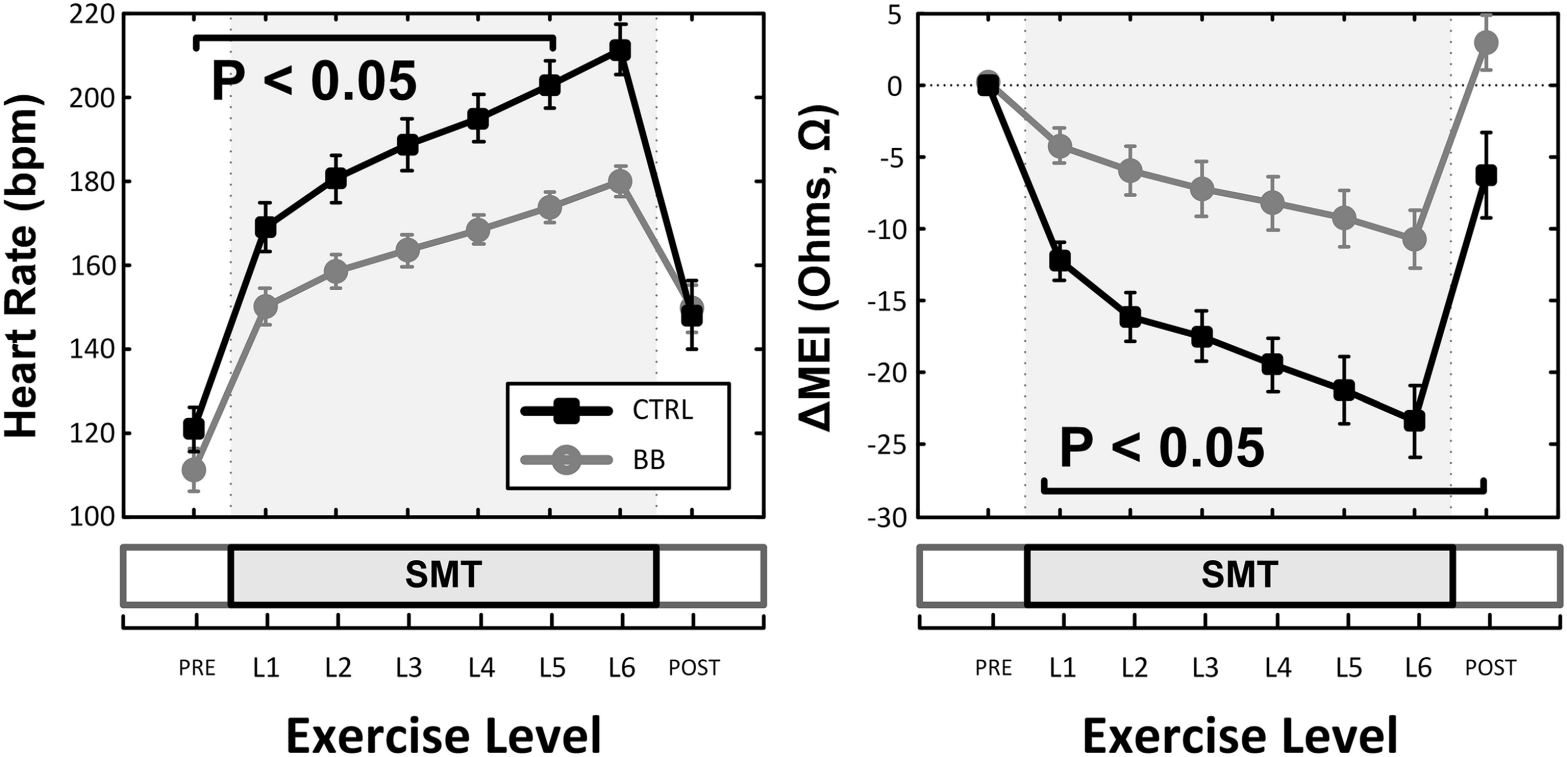
**Effects of (non-selective) β-adrenoceptor blockade (+BB, propranolol) in the myocardial electrical impedance (MEI, right), and heart-rate (HR, left) response(s) to exercise; β-adrenoceptor blockade blunted the MEI response to exercise**. SMT, submaximal exercise test.

**Figure 5:**
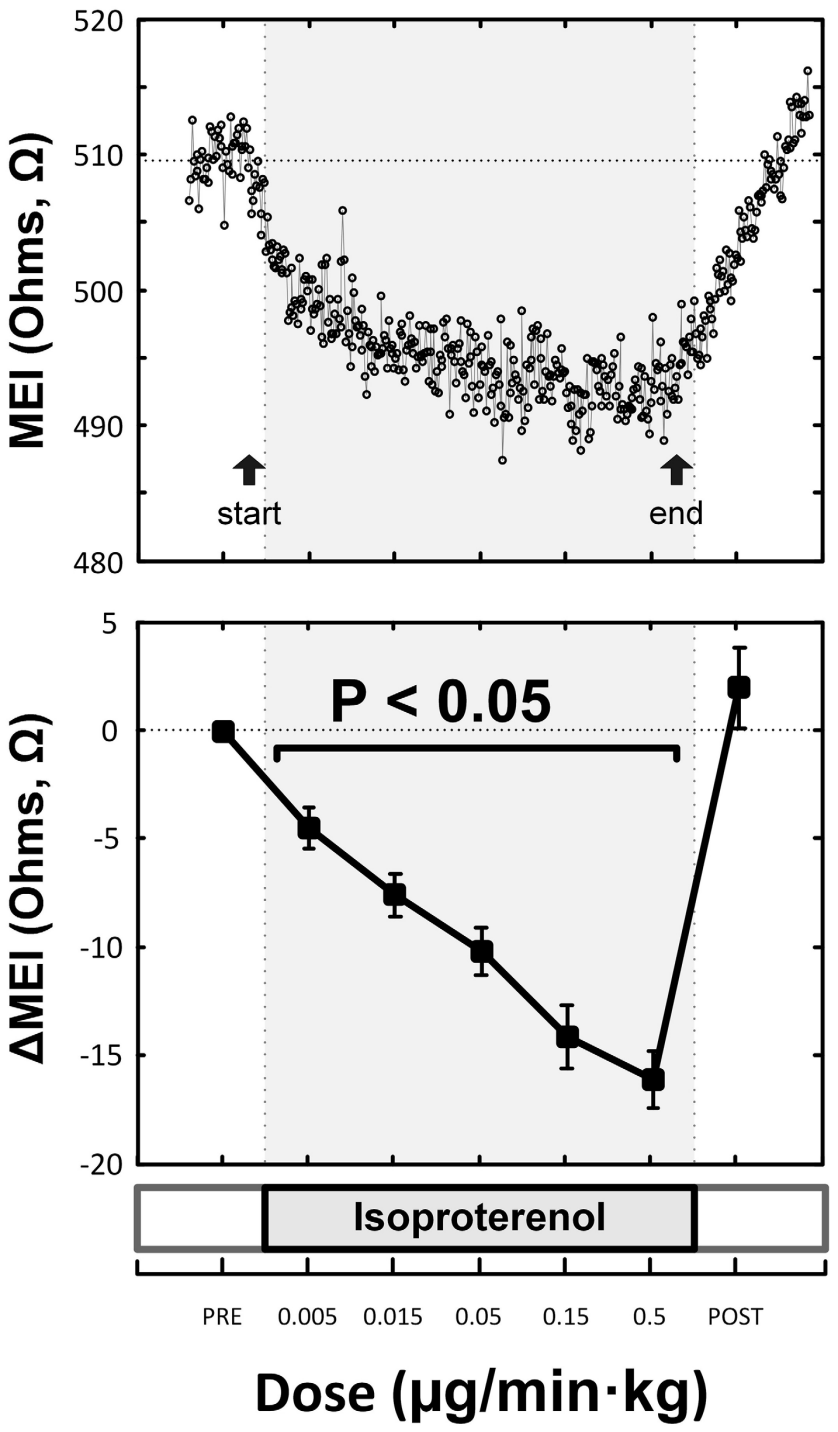
**Representative (top) and overall/mean (bottom) myocardial electrical impedance (MEI) response to direct β-adrenoceptor stimulation at rest (via escalating-dose infusion of isoproterenol), showing dose-dependent MEI decrease**.

Indeed, pretreatment with the (non-selective) β-AR antagonist propranolol significantly attenuated the MEI response to exercise (e.g., at L6, CTRL: −23 ± 2.5 Ω vs. BB: −11 ± 2.0 Ω; *P* < 0.05, *n* = 15) (see Figure 4, Table 2), markedly reducing the slope of the ΔMEI vs. ΔHR (−0.250 vs. −0.139 Ω /bpm; *R*^2^ = 0.78, *P* < 0.05) and the ΔMEI vs. ΔVT relationships (Ω /ln(ms^2^); *R*^2^ = 0.77, *P* < 0.05). Similarly, as expected, β-AR blockade blunted the exercise-induced heart rate increase (e.g., at L6, CTRL: +47 ± 6 vs. BB: +30 ± 5 bpm; see Figure 4), but accentuated cardiac parasympathetic withdrawal (e.g., lower vagal tone index values were recorded). Moreover, direct β-adrenoceptor stimulation at rest (with isoproterenol infusions) triggered a dose-dependent MEI response (decrease) comparable to that observed during submaximal exercise (see Figure 5). On average, at the highest dose-level assayed (i.e., at 0.5 μg/min·kg), isoproterenol decreased MEI by −14 ± 1.7 Ω (from 453 ± 40 to 440 ± 45Ω, *P* < 0.05). Thus, when considered together, these data suggest that the acute electrotonic (impedance) changes induced by exercise are predominantly mediated by sympathetic β-adrenoceptor activation.

**Table 2:**
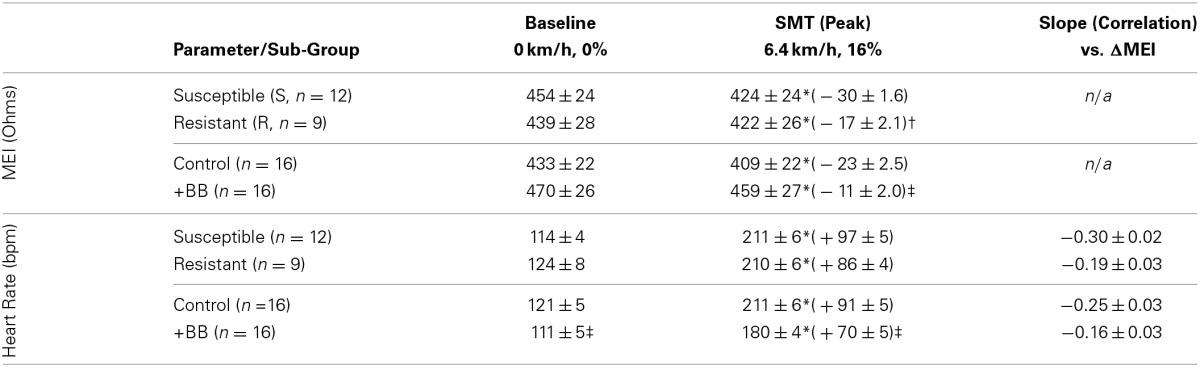
**Exercise-induced changes in both the myocardial electrical impedance (MEI) and the heart-rate (HR) of awake-unsedated dogs with healed left-anterior descending (LAD) myocardial infarct; comparative effects of the underlying susceptibility to malignant arrhythmias (S vs. R), and of β-adrenoceptor blockade (+BB)**.

^*^P < 0.05 vs. Baseline.

^†^P < 0.05 vs. Susceptible.

^‡^P < 0.05 vs. Control.

**Figure 6:**
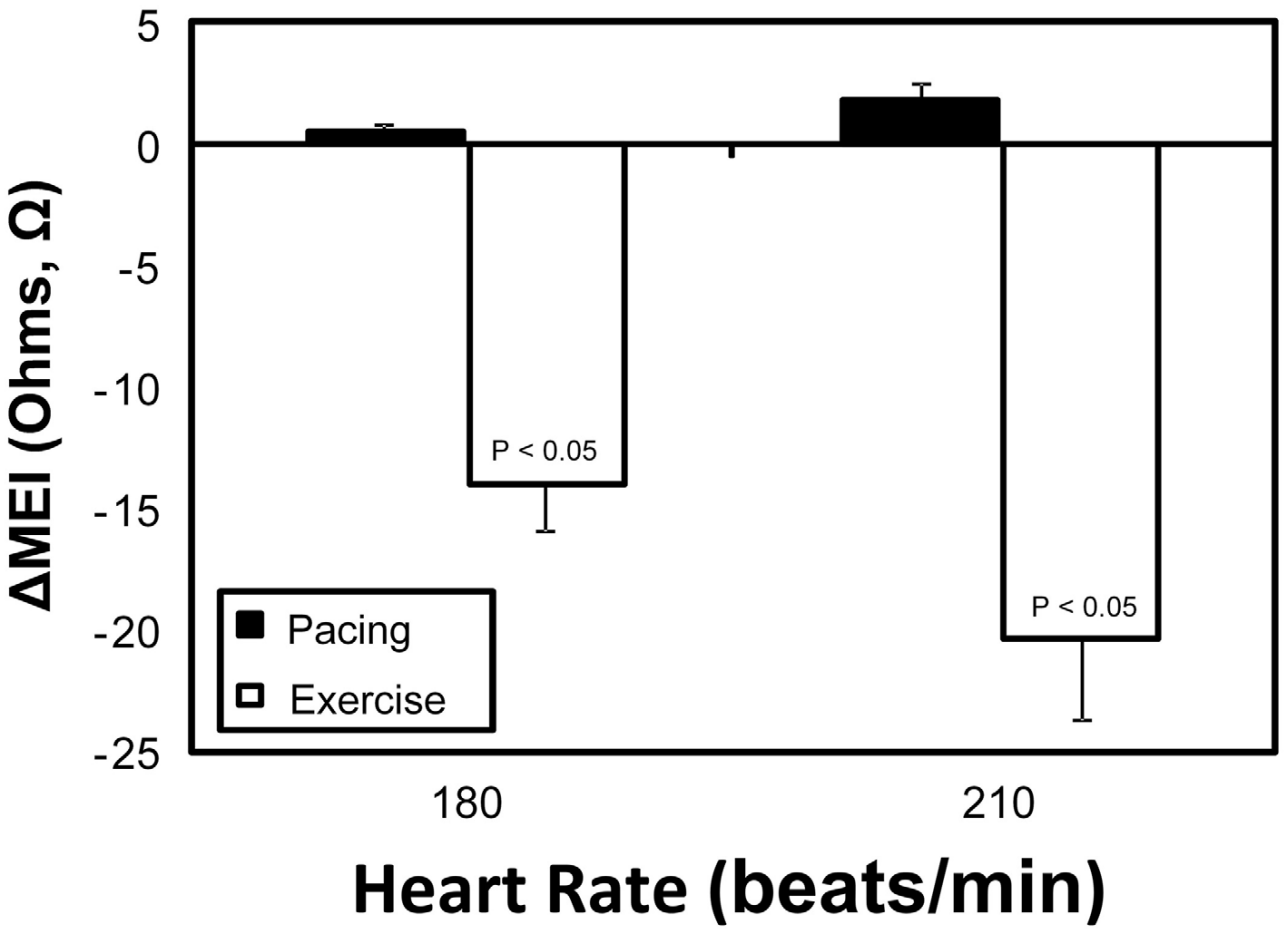
**Comparative myocardial electrical impedance (MEI) response to either submaximal exercise (white) or acute rate-matched ventricular pacing (black); only exercise decreased MEI**.

On the other hand, while both submaximal exercise and direct β-adrenoceptor stimulation (at rest) led to acute MEI reductions, suggestive of favored increased electrotonic coupling, elevations in heart-rate via overdrive left-ventricular pacing had no significant effects on the passive electrical properties of the myocardium (see Figure 6). For instance, when resting animals were paced at 210 beats/min (from a basal rate of 123 ± 7 bpm) MEI changed only +2 ± 0.8Ω (from 426 ± 28Ω at baseline to 428 ± 29Ω, *n* = 10; N.S.); meanwhile, when the same subset of animals performed a submaximal exercise test, MEI decreased −20 ± 3.3Ω (from 435 ± 38Ω at rest to 414 ± 38.3Ω at L6, *n* = 8; *P* < 0.05) although heart rate increased similarly (from 124 ± 7 at rest to 213 ± 9 bpm at L6, *P* < 0.05). These data support the conclusion that the effects of exercise on electrotonic coupling (enhancement) are not mediated by its concomitant chronotropic effects (i.e., rate acceleration).

### Arrhythmia susceptibility

**Figure 7:**
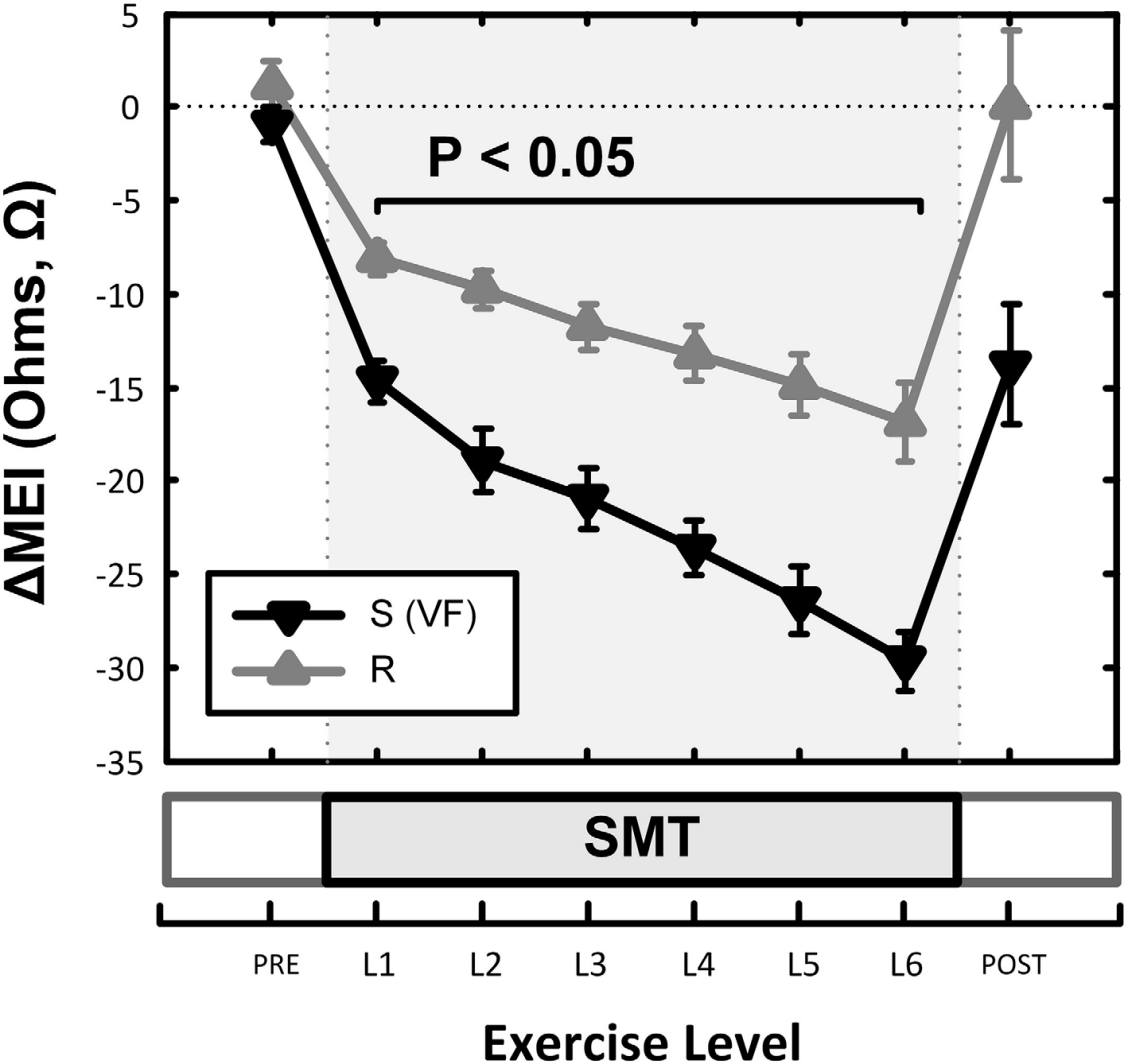
**Effects of underlying arrhythmic susceptibility of each animal in the myocardial electrical impedance (MEI) response to exercise; animals prone to ischemia-induced VF (i.e., S; *n* = 12) had a significantly larger MEI response to submaximal exercise, when compared those that were resistant (i.e., R; *n* = 9)**.

Although (as mentioned above), MEI decreased with exercise onset in all animals studied, the degree of reduction was modulated by the underlying arrhythmic susceptibility of each animal: Animals prone to ischemia-induced VF had a significantly larger MEI response to submaximal exercise (see Figure 7, Table 2). At the peak exercise level (i.e., at L6), for instance, MEI decreased −30 ± 1.6Ω in dogs susceptible to VF (S, *n* = 12) and only −17 ± 2.1Ω in those resistant (R, *n* = 9) (*P* < 0.05), albeit comparable heart rates (e.g., S: 211 ± 5 vs. R: 210 ± 6 bpm at L6, N.S.) and heart rate variability indices (e.g., VT; S: 1.3 ± 0.3 vs. R: 0.7 ± 0.2 ln ms^2^ at L6, N.S.) were reached by both groups; these observations suggest an increased impedance responsiveness to exercise-induced autonomic neural activation in dogs prone to arrhythmias.

In fact, susceptible animals had significantly (*P* < 0.001, see Table 2) steeper relationships between the exercise-mediated changes in impedance (ΔMEI) and those recorded for the heart rate (slope of ΔMEI vs. ΔHR; S: −0.295 vs. R: −0.180 Ω/bpm, *R*^2^ = 0.87) and/or the vagal tone index (slope of ΔMEI vs. ΔVT; S: 4.11 vs. R: 2.09 Ω/ln ms^2^, *R*^2^ = 0.85). Notably, such marked intergroup differences were evident even when the slopes of the ΔMEI vs. ΔHR (S: −0.31 ± 0.2 vs. R: −0.18 ± 0.2 Ω/bpm, *P* = 0.001) and/or the ΔMEI vs. ΔVT (S: 4.3 ± 0.27 vs. R: 2.4 ± 0.46 Ω/ln ms^2^, *P* = 0.002) relationships were calculated individually for each animal, rather than from the study groups. Moreover, complete β-AR blockade (with propranolol) blunted the exercise-induced impedance differences between animals susceptible (S, *n* = 7) and resistant (R, *n* = 5) to ischemia-induced arrhythmias (during control test; S: −30 ± 2.4 vs. R: −17 ± 2.7 Ω, *P* < 0.05, but after β-AR blockade; S: −16 ± 1.5 vs. R: −10 ± 4.1Ω, N.S.).

### Electrocardiographic data (response to exercise)

Concomitantly with the above mentioned changes on indices of autonomic neural activation (cardio-acceleration, decreased heart-rate variability), exercise shortened the PR-interval and flattened the T-wave (data not shown) while decreasing indices of ventricular repolarization temporal duration (QTc, TPE) (see Table 3). For instance, on average, exercise shortened the rate-corrected QT-interval from 251 ± 4 msc at rest to 229 ± 4 msc (*P* < 0.05), suggesting a faster and/or more homogeneous repolarization. On the other hand, exercise increased the QT/TQ ratio (an index of the steepness of ventricular restitution, Fossa et al., 2007; Kijtawornrat et al., 2010) as well as the standard deviation of the T-wave amplitude (a marker of repolarization variability and/or T-wave “alternans”). Notably, exercise-induced changes on the T-wave amplitude variability were larger in animals susceptible to ischemia-induced VF (from 20.5 ± 3.1 μ V at rest to 50.5 ± 9.0 μ V post-exercise, *P* < 0.05) than in those resistant such arrhythmias (from 25.8 ± 4.7 μ V at rest to 39.2 ± 5.6 μ V post-exercise, N.S.); no other significant electrocardiographic differences, either at rest or following exercise, were noted between animals susceptible and resistant to VF.

**Table 3:** **Electrocardiographic response(s) to submaximal exercise**.

**Time**	**RR(ms)**	**PR (ms)**	**QRS (ms)**	**QT (ms)**	**QT_**c**_ (msc)**	**TPE (ms)**	**QT/TQ (n/u)**	**T_**SD**_ (μV)**
*Baseline*	479 ± 19	92 ± 3	66 ± 2	206 ± 5	251 ± 4	51 ± 4	0.79 ± 0.04	25 ± 3
*Exercise*	329 ± 8	79 ± 3	65 ± 3	170 ± 4	229 ± 4	39 ± 2	1.11 ± 0.06	45 ± 5
***P < 0.05***^†^	↓	↓	−	↓	↓	↓	↑	↑

Data collected immediately after the discontinuation of exercise.

† Arrows (↓,↑): P < 0.05 Exercise vs. Baseline (rest).

QTc, van der Water's rate-corrected QT interval; TPE, Tpeak-Tend; QT/TQ, index reflecting the steepness of restitution; TSD, Standard deviation of the T-Wave amplitude.

## Discussion

The present study investigated the acute effects of submaximal exercise and its resulting autonomic neural activation on the myocardial electrotonic coupling of dogs with healed myocardial infarctions, as described by changes in the electrical impedance of surviving remote (i.e., non-infarcted) myocardium. This study demonstrated that acute β-adrenoceptor activation (either during bouts of exercise or via a direct pharmacological challenge) acutely increased passive (electrotonic) coupling in the myocardium, with the largest changes noted in those animals that were subsequently shown to be susceptible to malignant ventricular arrhythmias. In short, myocardial impedance was shown to decrease gradually as the level of exercise and autonomic neural activation increased. In contrast, ventricular overdrive pacing (at heart rates matched to those seen with exercise) had no significant effects on MEI.

As noted by Kléber et al. (1987), the gross myocardial electrical impedance measurements used in this study as a surrogate of electrotonic coupling represent the combined passive electrical properties of the intra-, extra- and inter- (i.e., junctional) cellular pathways. Thus, the observed changes (i.e., decreases) in impedance can be a reflection of both direct changes (increases) in cell-to-cell coupling at the gap-junctions, and/or geometrical effects affecting the myocyte/interstitial space ratios during exercise (Fleischhauer et al., 1995; De Mello, 2010) (see below). Notably, pharmacological interventions that result in stimulation of the β-AR/adenylyl-cyclase/PKA pathway and increase cAMP concentration have been shown to enhance gap junctional coupling in many preparations (e.g., see Manoach et al., 1995; De Mello, 1996a; Dhein, 2004). In agreement with these *in vitro* reports, complete β-AR blockade attenuated the MEI response to exercise while direct β-AR stimulation (at rest with isoproterenol) triggered MEI decreases comparable to those observed during exercise.

Moreover, in the present study, β-AR meditated exercise-induced electrotonic changes were not only demonstrated *in vivo* (conscious animals), but more importantly, the magnitude of these changes were shown to differ between post-MI dogs subsequently shown to be susceptible or resistant to ischemia-induced ventricular fibrillation (VF). In this clinically-relevant scenario, a significantly larger MEI response to exercise was noted in animals prone to malignant arrhythmias (i.e., VF), suggesting increased electrotonic responsiveness to autonomic (β-AR) activation. The VF-susceptible animals have been extensively studied, presenting marked derangements in ionic current (Sridhar et al., 2008; Bonilla et al., 2012), electrotonic coupling (Del Rio et al., 2008b), intracellular calcium homeostasis (Belevych et al., 2009, 2012), and autonomic control (see Billman, 2006). For instance, susceptible dogs have been shown to have a larger degree of electrotonic remodeling post-MI (Del Rio et al., 2008b), presenting moderately (albeit not statistically significant) higher remote MEI values at rest which could favor the observed increased electrotonic responsiveness. Indeed, in this study, animals prone to arrhythmias tended to have slightly higher baseline (i.e., pre-exercise) impedances but reached similar (absolute) values during exercise, suggesting, perhaps, a role of basal electrotonic derangements to their increased passive electrical responses to β-AR activation. Interestingly, the post-MI remodeling (e.g., down-regulation) of junctional proteins mediating electrotonic coupling is a well-established risk factor for malignant arrhythmias (e.g., see Saffitz and Kléber, 2012). On the other hand, susceptible animals also exhibit enhanced cardiac β-AR responsiveness, presenting a dominant functional contribution of the β_2_-adrenergic receptors following MI, both *in vivo* and *in vitro* (Billman et al., 1997; Houle et al., 2001). Notably, acute β_2_-AR stimulation can increase junctional conductance and protein expression in cardiac myocytes (Xia et al., 2009), likely via the exchange protein directly activated by cAMP (Epac)/Rap1 signaling pathway (Somekawa et al., 2005; Duquesnes et al., 2010; Mostafavi et al., 2014).

It also should be noted that much like patients prone to SCD (Rubart and Zipes, 2005), post-MI VF-susceptible dogs have well-documented abnormalities in myocardial calcium handling, being characterized by leaky and oxydized ryanodine receptors (Belevych et al., 2009) as well as increased calcium (Ca^2+^) entry and Ca^2+^ transients, particularly during β-AR stimulation (Billman et al., 1997; Altschuld and Billman, 2000; Belevych et al., 2012). Moreover, in these animals, β_2_-AR mediated increases in Ca^2+^-transient amplitudes and Ca^2+^-entry have been reported (Billman et al., 1997), rendering them exceptionally responsive (i.e., anti-arrhythmic protection) to the pharmacological blockade of L-type current (Billman, 1989). Interestingly, intracellular Ca^2+^ and junctional/electrotonic conductance are tightly coupled. For instance, inhibition of junctional communication can attenuate Ca^2+^ transients and sparks (Li et al., 2012). Meanwhile, pathologically-elevated intracellular Ca^2+^ levels (e.g., during a sustained ischemic insult) have been shown to decrease junctional conductance leading to electrotonic uncoupling (Cascio et al., 1990, 2005; Kléber, 1992; Smith et al., 1995; Owens et al., 1996; García-Dorado et al., 2004; De Groot and Coronel, 2004), likely as a protective mechanism against the spread of calcium overload (i.e., myocytes live and work together but die alone; quote from Engelmann (1875) in Janse et al., 2002). In contrast, moderate (i.e., within physiological levels) intracellular Ca^2+^ changes can enhance junctional coupling (perhaps via Ca^2+^-activated kinases) (e.g., see Delage and Délèze, 1999). Indeed, Joyner et al. (1996) using a hybrid (both *in vitro and in silico*) paired-myocyte model showed that both β-AR stimulation (with isoproterenol) and direct opening of the L-type Ca^2+^ channels (with Bay K8644), facilitated cell-to-cell coupling and impulse propagation (an effect prevented by the L-type Ca^2+^ channel antagonist nifedipine). Thus, β-AR mediated electrotonic changes can provide mechanistic explanation(s) for the enhanced electrotonic responsiveness observed in the present study, particularly when the fact that complete β-AR blockade abolished the exercise-induced (electrotonic) differences with their VF-resistant counterparts is considered.

### Clinical implications

Regardless of the potential mechanism(s), the observed increase in electrotonic coupling during exercise and/or β-adrenergic autonomic activation may have important clinical implications. Notably, enhancements in electrotonic coupling can reduce repolarization heterogeneities thereby blunting or masking intrinsic pro-arrhythmic ionic substrates. Indeed, increased electrotonic coupling has been shown to suppress early after-depolarizations (EADs) and reduce pro-arrhythmic dispersion of repolarization (e.g., Huelsing et al., 2000; Quan et al., 2007; Himel et al., 2013). Interestingly, Vanoli et al. (1995) showed that adrenergic activation via either left stellate ganglion stimulation (*in vivo*) or isoproterenol administration (*in vitro*) blunted the d-sotalol-mediated prolongation of the action potential duration. Similarly, Järvenpää et al. (2007) reported that post-MI patients susceptible to VF have an impaired capacity of the autonomic nervous system to alter (i.e., prolong) electrocardiographic indices of repolarization. These observations are in agreement with the results of the present study; namely, the observed shortening of both the rate-corrected QT interval and the terminal portion of the T-wave (reflecting spatial repolarization heterogeneities); changes that are consistent with the exercise-mediated increases in myocardial electrotonic coupling.

Finally, substantial evidence supports the role of abnormal repolarization and calcium mishandling during neural autonomic activation in the onset and maintenance of lethal arrhythmias, particularly in the setting of both congenital and acquired (e.g., post-MI) electro-mechanical remodeling. For instance, exercise testing has been shown to amplify the arrhythmic genotype-phenotype relationship in patients with both long-QT syndrome (Takenaka et al., 2003) and catecholaminergic polymorphic ventricular tachycardia (Obeyesekere et al., 2011). However, in a stark contrast, exercise-driven risk stratification of post-MI patients (particularly those with preserved ejection fraction) remains difficult and, at times, counter-intuitive. For instance, in a recent meta-analysis, Chan et al. (2010) showed that abnormal microvolt TWA results were more likely to reflect an increased risk for arrhythmic events only when β-adrenoceptor blocker therapy was not withheld prior to testing. In a similar manner, cardiac pacing elicited not only positive TWA responses in susceptible patients (Hohnloser et al., 1997; Raatikainen et al., 2005), but led to a lower incidence of indeterminate test results when compared to exercise testing (Kraaier et al., 2009). In this study, exercise not only shortened the Tpeak to Tend interval (TPE), an index that has been reported to prolong during pro-arrhythmic stimulation (Johnson et al., 2013), but failed to induce significant TWA differences between susceptible and resistant animals (i.e., S: 50.5 ± 9.0 vs. R: 39.2 ± 5.6 μ V, N.S.). Thus, enhanced myocardial electrotonic coupling mediated by exercise-induced β-adrenoceptor activation could mask (e.g., via reductions in repolarization heterogeneities) changes in indices of risk for arrhythmia and could thereby explain the false negative results often obtained by exercise stress testing.

On the other hand, it is also important to note that improved electrotonic coupling has been shown to blunt myocardial ionic heterogeneities (e.g., Huelsing et al., 2000; Quan et al., 2007; Himel et al., 2013), masking and even reducing pro-arrhythmic risk (e.g., via pharmacological gap-junction modulation,; Hennan et al., 2006; Kjølbye et al., 2008). However any improvement in electrotonic coupling that was induced by acute exercise was of insufficient magnitude to prevent the onset of ischemic (coronary artery occlusion) arrhythmias in susceptible animals. Indeed, myocardial ischemia has been shown to depress electrotonic coupling acutely (e.g., Kléber et al., 1987; Cascio et al., 1990; Smith et al., 1995; Del Rio et al., 2005, 2008a). Thus, in the setting of a healed infarction/remodeling (Del Rio et al., 2008b) and exercise-mediated electrotonic enhancements, concomitant regional ischemia likely resulted in marked local passive electrical heterogeneities, which are pro-arrhythmic (e.g., Bishop et al., 2014). Similarly, as noted above, impedance measurements represent the “average” electrotonic properties, (Kléber et al., 1987) of a specific myocardial region. Therefore, the observed MEI decreases are unlikely to reflect a homogenous enhancement of electrotonic coupling. Indeed, the heterogeneous distribution of the myocardial autonomic innervation, would favor the onset of “focal” arrhythmias during β-AR stimulation (Myles et al., 2012).

### Study limitations

This study demonstrated that β-adrenoceptor activation mediates exercise-driven changes (increased) in myocardial electrotonic coupling. However, it also should be noted that despite complete β-adrenoceptor blockade, a moderate decrease in MEI was observed during exercise. Several factors, that were not assessed in the present study, may have contributed to the residual (non- β-AR mediated) increases in myocardial electrotonic coupling during exercise.

For instance, during exercise, circulating cathecholamines activate both β- and α-adrenoceptors. Interestingly, sub-chronic α-adrenergic stimulation has been reported to exert PKC-mediated enhancements in connexin43 expression (Salameh et al., 2006). Furthermore, Rojas-Gomez et al. (2008) found that phenylephrine (an α-adrenergic receptor agonist) enhanced Cx43 expression in neonatal rat cardiac myocytes, resulting in enhanced gap-junction conductance. However, it also should be noted that opposite electrotonic (i.e., reductions in junctional conductance) have been reported during acute α-adrenoceptor stimulation (De Mello, 1997; de Boer et al., 2007), albeit these effects may vary in the setting of concomitant β-adrenoceptor stimulation (Salameh et al., 2010). Similarly, the renin-angiotensin system is also acutely activated during exercise, increasing the circulating levels of angiotensin-II, which can modulate the passive electrical properties of the myocardium (De Mello, 1996b, 2014; Sovari et al., 2013).

Also, as noted above, changes in both spatial/geometrical and/or ionic composition of the myocardium can alter its electrotonic properties. For example, Veeraraghavan et al. (2012) recently reported that changes in interstitial volume can modulate both conduction velocity and its dependence on gap-junction conductance. Changes in interstitial and/or myocyte volumes during exercise are likely, particularly given the reported ionic, metabolic, and plasma-volume changes during exercise (Paterson, 1996); interestingly, β-AR activation has been implicated in the volume-regulation (i.e., decrease) of cardiac myocytes (Wang et al., 1997). Thus, β-adrenoceptor mediated changes in myocyte volume could also contribute to the exercise-induced changes MEI reported in the present study.

Similarly, changes in both cell-to-cell coupling (e.g., Burt and Spray, 1988; Saffitz and Yamada, 1998; Salameh and Dhein, 2013) and global indices of myocardial passive electrical properties (e.g., Sasaki et al., 1994; Dekker et al., 1996; Howie et al., 2001) have been linked/associated with alterations in the mechanical properties of the myocardium. Indeed, in isolated myocytes, positive/negative inotropic agents have been shown to enhance/depress (respectively) junctional coupling in parallel with their functional effects (Burt and Spray, 1988; Dhein, 2004). In the present study, both exercise and direct pharmacological β-AR stimulation (with isoproterenol), two well-defined inotropic interventions, decreased MEI, consistent with an enhanced electrotonic coupling. Although neither systemic/cardiac hemodynamics nor mechanics were assessed directly in the present study, previous studies have extensively documented the hemodynamic/functional responses of susceptible/resistant post-MI dogs, both at rest and during exercise (e.g., Billman et al., 1985, 1997; De Ferrari et al., 1993; Avendano and Billman, 1994). For instance, Billman et al. (1985) reported comparable increases in the peak rate of left-ventricular pressure change (i.e., dP/dtmax—an inotropic index) during exercise in both groups of post-MI dogs, with animals prone to arrhythmias showing larger elevations in ventricular filling (end-diastolic) pressures (consistent with the exercise-driven preload changes, Miyazaki et al., 1990) and blunted increases in systolic pressures. Meanwhile, Avendano and Billman (1994) described similar inotropic/hemodynamic changes (i.e., dP/dtmax and systolic pressure increases) following isoproterenol administration (and other interventions that increased cAMP levels) in post-MI dogs and, in contrast to exercise, moderate isoproterenol-mediated end-diastolic pressure reductions were also noted (consistent with pre-load reductions Barnes et al., 1979). Hence, as mechanical stretch can alter intercellular coupling (Salameh and Dhein, 2013), it is possible that exercise-mediated changes in myocardial wall-tension could also play a role in the passive electrical changes that were observed in the present study. Finally, neither myocardial nor core body temperature were measured during exercise in this study. Notably, the resistivity of a medium can decrease as temperature increases (e.g., 2%/°C; Tsai et al., 2002). However, previously unpublished data from our laboratory (Billman GE, and del Rio CL) found that core body temperature not only increased moderately (~1°C) during exercise, but also, recovered slowly post-exercise (consistent with the limited heat-dissipation of dogs); an observation that contrasts with the rapid restoration of electrotonic coupling following the termination of exercise. Thus, changes in myocardial temperature induced by exercise probably did not contribute to the MEI changes noted in the present study.

In conclusion, the results of the present study demonstrate that β-AR activation during exercise can acutely enhance passive electrical properties (i.e., electrotonic coupling) of the myocardium, particularly in post-MI dogs susceptible to ischemia-induced VF. Increased coupling during β-AR stimulation may have important clinical implications, as it could mask intrinsic (and/or acquired) pro-arrhythmic repolarization abnormalities during states of autonomic activation (e.g., exercise) *in vivo*.

### Conflict of interest statement

The authors declare that the research was conducted in the absence of any commercial or financial relationships that could be construed as a potential conflict of interest.
